# HALF: Histogram of Angles in Linked Features for 3D Point Cloud Data Segmentation of Plants for Robust Sensing

**DOI:** 10.3390/s25123659

**Published:** 2025-06-11

**Authors:** Hidenori Takauji, Naofumi Wada, Shun’ichi Kaneko, Takanari Tanabata

**Affiliations:** 1 Department of Electronics and Information Engineering, Faculty of Engineering, Hokkai-Gakuen University, Sapporo 0640926, Japan; 2 Department of Information Science and Technology, Faculty of Information Science and Technology, Hokkaido University of Science, Sapporo 0068585, Japan; wada-n@hus.ac.jp; 3 Graduate School of Information Science and Technology, Hokkaido University, Sapporo 0640814, Japan; kaneko.s.0713@gmail.com; 4 Facility for Genome Informatics, Kazusa DNA Research Institute, Chiba 2920818, Japan; tanabata@kazusa.or.jp

**Keywords:** low-cost plant segmentation, soybean point cloud data, plant phenotyping, histogram, robust sensing

## Abstract

This paper presents a novel method, Histogram of Angles in Linked Features (HALF), designed for the segmentation of 3D point cloud data of plants for robust sensing. The proposed method leverages local angular features extracted from 3D measurements obtained via sensing technologies such as laser scanning, LiDAR, or photogrammetry. HALF enables efficient identification of plant structures—leaves, stems, and knots—without requiring large-scale labeled datasets, making it highly suitable for applications in plant phenotyping and structural analysis. To enhance robustness and interpretability, we extend HALF to a convolution-based mathematical framework and introduce the Sequential Competitive Segmentation Algorithm (SCSA) for phytomer-level classification. Experimental results using 3D point cloud data of soybean plants demonstrate the feasibility of our method in sensor-based plant monitoring systems. By providing a low-cost and efficient approach for plant structure analysis, HALF contributes to the advancement of sensor-driven plant phenotyping and precision agriculture.

## 1. Introduction

Plants survive in their environment by adapting to it. The knowledge obtained from analyzing gene functions related to environmental adaptation has been useful for breeding species that can adjust to their agricultural production locations and for improving species’ resistance to climate change [1]. To analyze gene functions, it is essential to study various genotypes under diverse growing conditions, clarify variations in individual growth, and quantify these differences using large-scale datasets. Recently, several studies have focused on the geometric analysis of plant parts, such as leaves and stems, and their structures in relation to size and shape [2,3].

Three-dimensional shapes represented by, for instance, 3D point cloud data are fundamentally important due to their generality and universality, along with the popularity of sensing technologies. With the rapid advancement of sensing technologies such as 3D laser scanning, LiDAR, and multi-view photogrammetry, large-scale three-dimensional (3D) point cloud data acquisition has become increasingly feasible in plant science. These sensor-based measurements play a crucial role in plant phenotyping, enabling non-destructive and high-throughput analysis of plant structures.

However, effectively segmenting such 3D data into meaningful plant organs remains a significant challenge, particularly in minimizing the need for manually labeled training data. Several machine learning-based methods, such as DCNNs, have similar objectives to ours but require a substantial amount of labeled training data. In our application of these methods to the analysis of living plants, which necessitates ongoing databases that include numerous individuals, the cost of preparing these data is a significant challenge; therefore, it is preferable to avoid any complicated processing if possible. For this reason, the novel method presented in this paper may be promising for cost savings. We develop an in-depth discussion on this issue in the following section.

We designed a fundamental and useful random algorithm, named HALF (Histogram of Angles in Linked Features), to transform their 3D point cloud data (PCD) into a labeled version using statistical features from an angular histogram based on a simple top-down definition of plant parts [4]. The algorithm is anticipated to be useful for the aforementioned purpose of phenotype analysis by examining the outer shape of plants. In this paper, we first propose some extensions of the basic tool to its theoretical version as a mathematical convolution operation. We then design a classification algorithm based on our HALF analysis for the complete segmentation of the PCD version of individual plants to facilitate phytomer extraction. Finally, we confirm the effectiveness of HALF through evaluation experiments using soybean PCD.

## 2. Related Works

Research on the segmentation of 3D point cloud data has been widely applied to the analysis and classification of plant structures. Conventional segmentation methods include approaches based on clustering and feature descriptors, which have been used extensively to analyze 3D point cloud data. For example, clustering-based methods can group data based on specific criteria, enabling the identification of plant geometries. However, clustering alone faces limitations in accurately distinguishing detailed differences in shapes and parts when applied to complex plant structures, thereby restricting segmentation accuracy.

Methods that combine handcrafted features and machine learning have also been utilized for plant segmentation using 3D point cloud data. Paulus et al. [5] proposed a method for classifying plant stems and leaves using PFH (Point Feature Histogram) [6] and FPFH (Fast Point Feature Histogram) [7] as feature descriptors. Similarly, Ziamtsov et al. [8] achieved high accuracy in distinguishing leaves and stems of tomatoes and tobacco by employing PFH, FPFH, SHOT (Signature of Histograms of Orientations) [9], and various machine learning methods. While these handcrafted features are effective for identifying basic plant structures, challenges remain regarding the preparation of labeled data and the high computational cost involved.

In recent years, semantic segmentation methods based on deep learning have advanced and are now being applied to the analysis of 3D point cloud data. Representative methods include PointNet [10] and PointNet++ [11], which directly extract features from input data and enable high-precision identification of plant organs such as leaves and stems. Patel et al. [12] and Luo et al. [13] applied PointNet and PointNet++ to plant phenotyping, achieving efficient organ-level segmentation. Additionally, Otobe et al. [14] successfully employed PointNet++ to separate leaves in the point cloud data of bell peppers, facilitating phenotype extraction.

Other methods have also been proposed. For instance, Boogaard et al. [15] introduced class-dependent sampling to address class imbalance, aiming to improve segmentation accuracy. Ghahremani et al. [16] combined a k-nearest neighbor algorithm with a deep learning model to identify the complex structures of wheat. Turgut et al. [17] used PointNet and PointNet++ to segment the organs of 3D models of roses, leveraging deep learning to identify plant organs. More recently, Xie et al. [18] applied Transformer-based methods such as Stratified Transformer and PAConv to plant phenotyping, utilizing self-attention mechanisms to enhance feature extraction. Research on point cloud deep learning remains active, and various segmentation techniques beyond PointNet and PointNet++ are being explored [19].

However, while these deep learning methods demonstrate high expressiveness in identifying plant organs, they require large amounts of labeled data, leading to significant costs associated with data preparation. The proposed HALF method differs from conventional segmentation techniques based on handcrafted features and deep learning. It focuses on angle histograms within 3D point cloud data to efficiently extract angle-specific characteristics unique to each plant part. This approach can identify plant parts without the need for large-scale labeled data, thus reducing data preparation costs. Additionally, HALF can be implemented through a simple algorithm based on randomness, requiring relatively low computational costs. This study demonstrates that the proposed HALF-based segmentation is effective for the efficient identification of plant structures. The contributions of this study are summarized as follows:

Low-cost shape identification: HALF does not require labeled data, allowing for easy application to large datasets at low cost.

Novel segmentation method using angle histograms: HALF efficiently extracts features useful for identifying plant parts.

Applicability to plant phenotyping: HALF offers a straightforward method for quantifying plant morphology, which is expected to benefit applications in plant phenotyping.

## 3. Fundamentals of HALF

### 3.1. Basic Definitions

To design effective features for phytomer segmentation, we can utilize natural characteristics such as shapes, sizes, colors, textures, and structures. In this paper, for three representative classes of plant parts—stems, knots, and leaves—we concentrate on their shapes as the targets of investigation because they are robust to changes in illumination and detectable at relatively low cost using effective applications of 3D tools, providing informative data for classification.

Histograms have a long history as an effective framework for representing and generating basic statistical features of various types of real-world data in many fields [20,21]. Their non-parametric nature has proven to be particularly effective in cases where sophisticated parametric statistical models cannot be utilized due to a lack of advanced knowledge about the targets or disturbances in observation.

**Figure 1:**
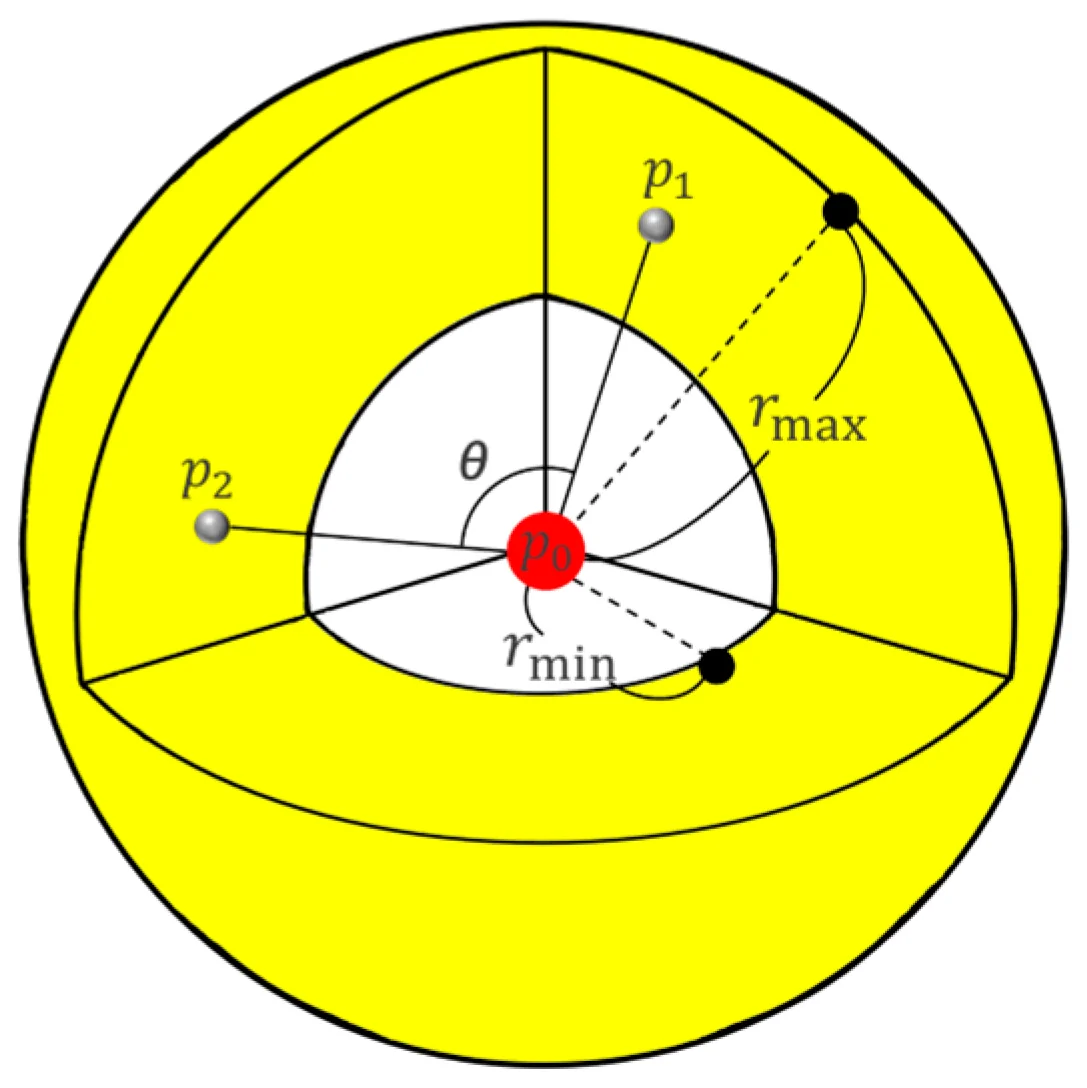
Shell for sampling three PCD points.

The main problem this paper addresses is how to determine the type of quantity that should be represented in histograms for effective classification, alongside an efficient and robust computational process. To this end, we propose the use of simple angular values between any pair of three-dimensional points, $p_{1}=\left(x_{1},y_{1},z_{1}\right)$ and $p_{2}=\left(x_{2},y_{2},z_{2}\right),$ centered around a nodal point, $p_{0}=\left(x_{0},y_{0},z_{0}\right)$. Each point has three-dimensional coordinates contaminated by independently and identically distributed random noise and is selected from point cloud data (PCD). Points should be chosen to have relatively longer lengths in both arms to better capture essential characteristics of part shapes, such as stems, knots, and leaves. We assume these coordinates are disturbed by normally distributed noise with zero mean and variance $\sigma_{0}^{2}$ originating from the 3D measurement process. Figure 1 illustrates a conditional selection of a tuple $\left(p_{1},p_{2}\right)$ around a center of interest $p_{0}$, where both elements lie in the gap or shell between two spheres of the radii $r_{min}$ and $r_{max}$.

**Figure 2:**
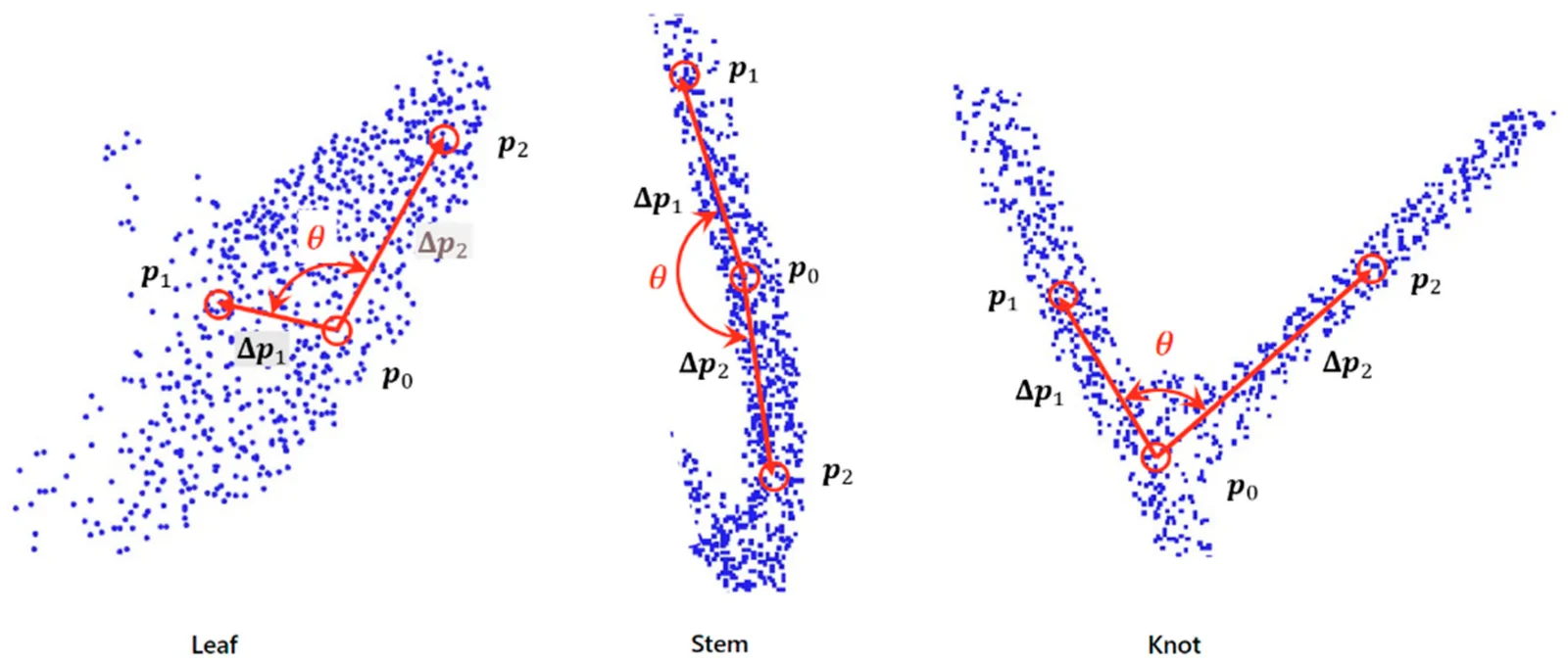
Representative structures of the three classes (Leaf, Stem, and Knot) in HALF analysis.

Figure 2 outlines the selection scheme proposed for typical shapes of stems and leaves.

After the initial selection process, two difference vectors—${\Delta p}_{1}=p_{1}-p_{0}$ and ${\Delta p}_{2}=p_{2}-p_{0}$—are computed, doubling the noise variance to ${2\sigma }_{0}^{2}$. These vectors are then normalized to form the unit vectors ${\Delta \tilde{p}}_{1}={\Delta p}_{1}/\left|{\Delta p}_{1}\right|$ and ${\Delta \tilde{p}}_{2}={\Delta p}_{2}/\left|{\Delta p}_{2}\right|$, each scaled to unit length. In the final step, the angle ξ between ${\Delta \tilde{p}}_{1}$ and ${\Delta \tilde{p}}_{2}$ is calculated by

$$\theta =arccos\left({{\Delta \tilde{p}}_{1}}^{T}{\Delta \tilde{p}}_{2}\right)$$

**Figure 3:**
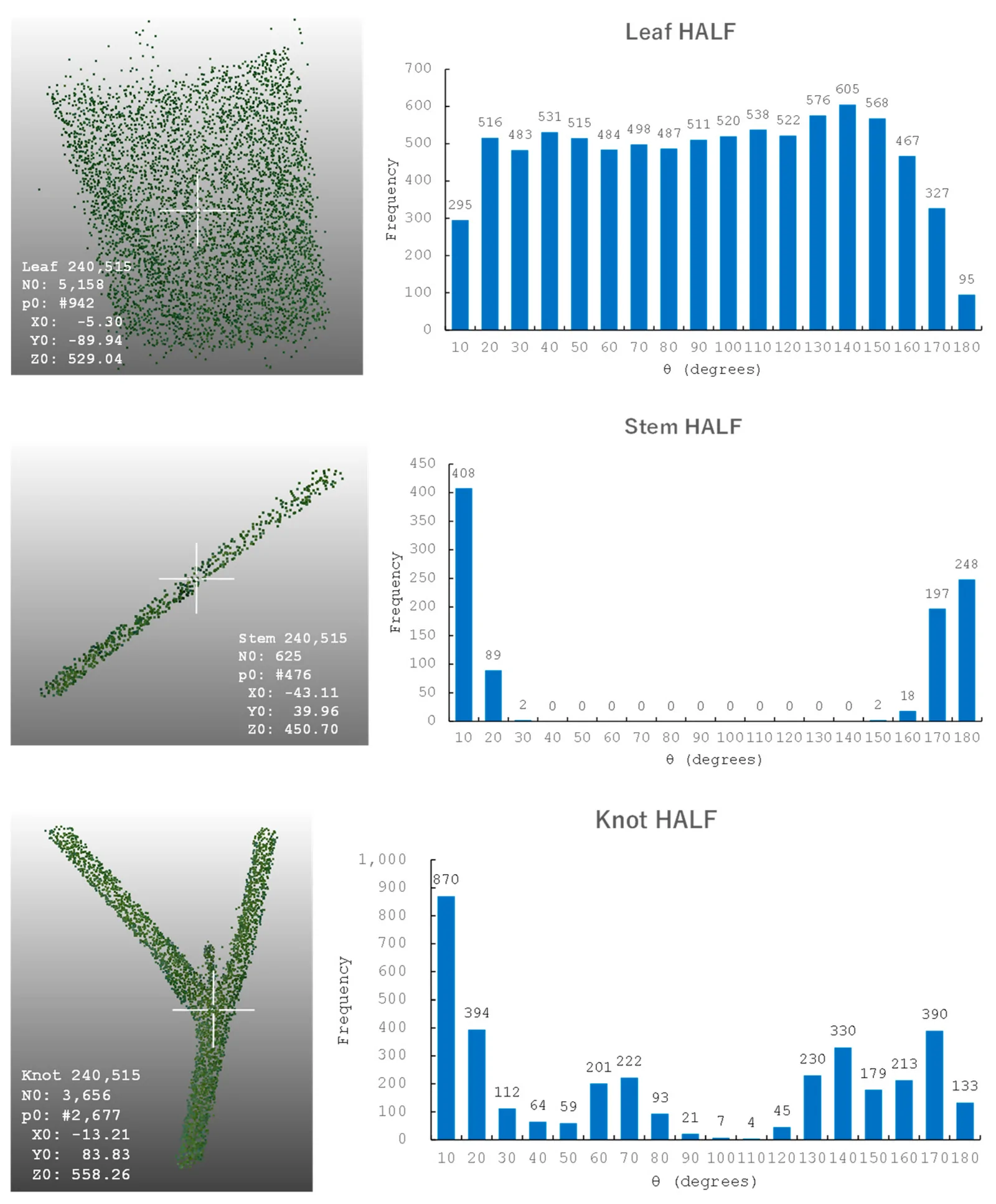
Examples of HALF for Leaf, Stem, and Knot classes.

For a fixed target point $p_{0}$ of interest, we aggregate the sampled angles θ for N pairs of ($p_{1}$, $p_{2}$) in the surrounding neighborhood into a histogram $H={\left\{f_{j}\right\}}_{j=1,\ldots ,p}$, where $f_{j}$ and p represent the frequencies in the j-th class and the total number of samples in the class, respectively. Figure 3 shows the HALFs for three classes with real PCD, demonstrating a certain degree of representation stability even with real data.

In this study, the parameters $r_{min}$ and $r_{max}$ shown in Figure 1 were set to 5 mm and 20 mm, respectively. These values were determined based on the actual sizes of soybean organs—such as stem diameter and internode spacing—and the point cloud density obtained in our measurement environment, in consultation with domain experts.

### 3.2. Convolution-Based Mathematics of HALF

**Figure 4:**
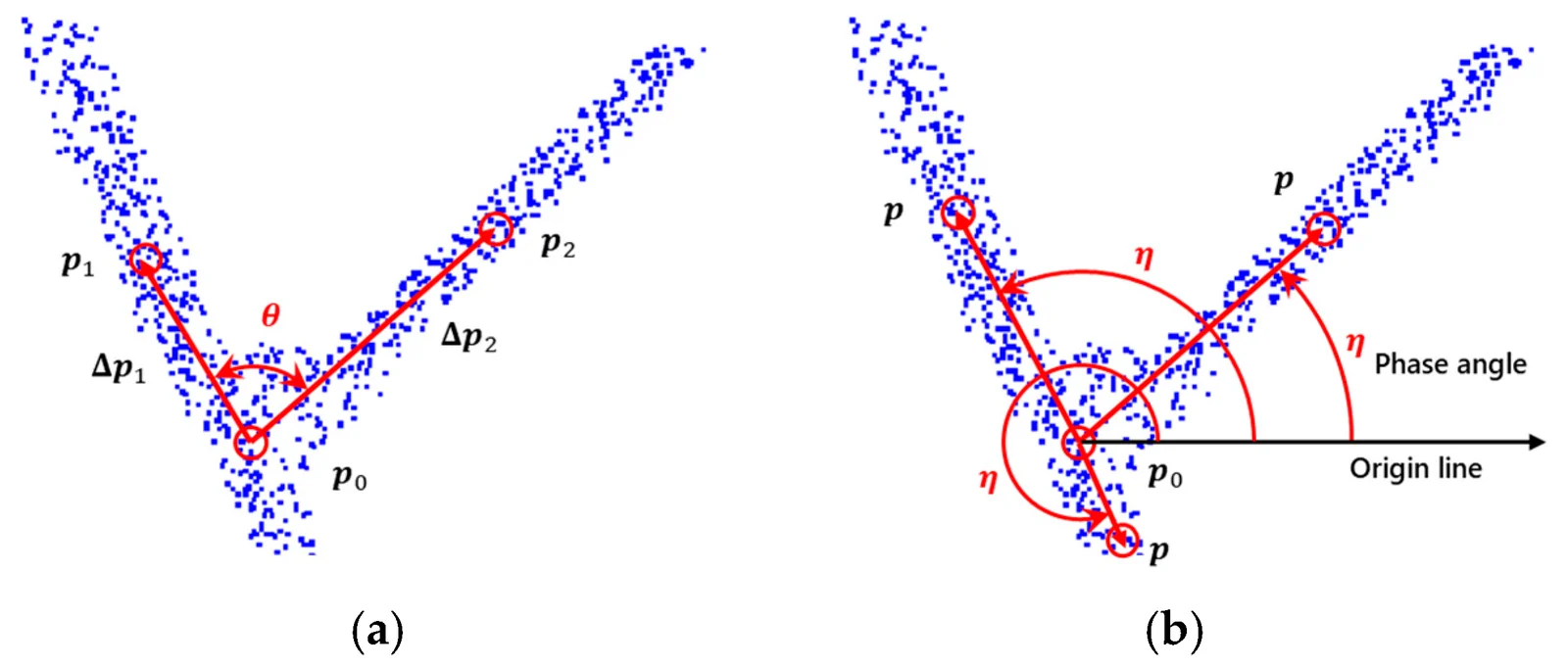
Intersection angle and phase angles. (**a**) Definition of an angle in HALF for Knot class (**b**) Definition of phase angles.

HALF is a statistic of angles between 3D point pairs $\left(p_{1},p_{2}\right)$ around an intentionally allocated interest point $p_{0}$ within the point set. We define the coordinate system just at $p_{0}$ and an axis or origin line directed towards an arbitrary orientation from the origin, while assuming statistical independence in the phase angle distributions of $p_{1}$ and $p_{2}$ around the origin $p_{0}$. When our objects are thick stems or leaves, they can be approximated by a geometrical line or plane. In these cases, the angle between two vectors $p_{1}-p_{0}={\vec{p}}_{1}$ and $p_{2}-p_{0}={\vec{p}}_{2}$ can always be represented by the difference between their respective phase angles of ${\vec{p}}_{1}$ and ${\vec{p}}_{2},$ as shown in Figure 4.

In these instances, any histogram of difference angles can be calculated by the convolving two histograms of phase angles [22].

Let us formalize the aforementioned concept. Figure 4a illustrates the definition of an interest point, a pair of selected points, and the angle between two vectors for a typical knot data set. This pair $\left(p_{1},p_{2}\right)$ establishes the intersection angle θ $\left(\in \left[0,\;180\right]\right)$ for HALF analysis. In Figure 4b the same angle can be represented as the difference between two phase angles η. A histogram of the phase angles is represented as follows:

$$H^{\eta }={\left\{f^{\eta }\left(\eta \right)\right\}}_{0\leq \eta <360}$$

Phase angle η is defined within the interval 0≤η<360. Intersection angle θ, which is essential for HALF analysis, is defined as the absolute value of the difference between two phase angles. Therefore, it can be calculated through the following three steps:

$$u=\eta_{1}-\eta_{2}$$

$$w=\left|u\right|$$

$$z=\left\{\begin{array}{c} w\;\;\;\;\left(0\leq w<180\right)\\ \;360-w\;\left(180\leq w<360\right) \end{array}\right.$$

where the three variables u, *w*, and *z* correspond to these three steps. The frequency function of *u* can be defined by the convolution of the two frequency functions of *$\eta_{1}$* and $\eta_{2}$ as follows:

$$f^{u}\left(u\right)=\sum_{\begin{array}{c} 0\leq \eta_{1}<360\\ 0\leq \eta_{2}<360 \end{array}}f^{\eta_{1}}\left(\eta_{1}\right)f^{\eta_{2}}\left(\eta_{2}\right)=\sum_{\begin{array}{c} 0\leq \eta_{1}<360\\ 0\leq \eta_{1}-u<360 \end{array}}f^{\eta_{1}}\left(\eta_{1}\right)f^{\eta_{2}}\left(\eta_{1}-u\right)$$

By transforming to absolute values and applying an operation at 180 degrees of the angle, their frequencies are defined by the functions as follows:

$$f^{w}\left(w\right)=f^{u}\left(w\right)+f^{u}\left(-w\right)$$

$$f^{z}\left(z\right)=f^{w}\left(z\right)+f^{w}\left(360-z\right)$$

The following are numerical examples of these mathematical expressions.

$$f^{u}\left(340\right)=f^{\eta_{1}}\left(340\right)f^{\eta_{2}}\left(0\right)+f^{\eta_{1}}\left(341\right)f^{\eta_{2}}\left(1\right)+\cdots +f^{\eta_{1}}\left(359\right)f^{\eta_{2}}\left(19\right)$$

$$f^{w}\left(200\right)=f^{u}\left(200\right)+f^{u}\left(-200\right)$$

$$f^{z}\left(42\right)=f^{w}\left(42\right)+f^{w}\left(318\right)$$

**Figure 5:**
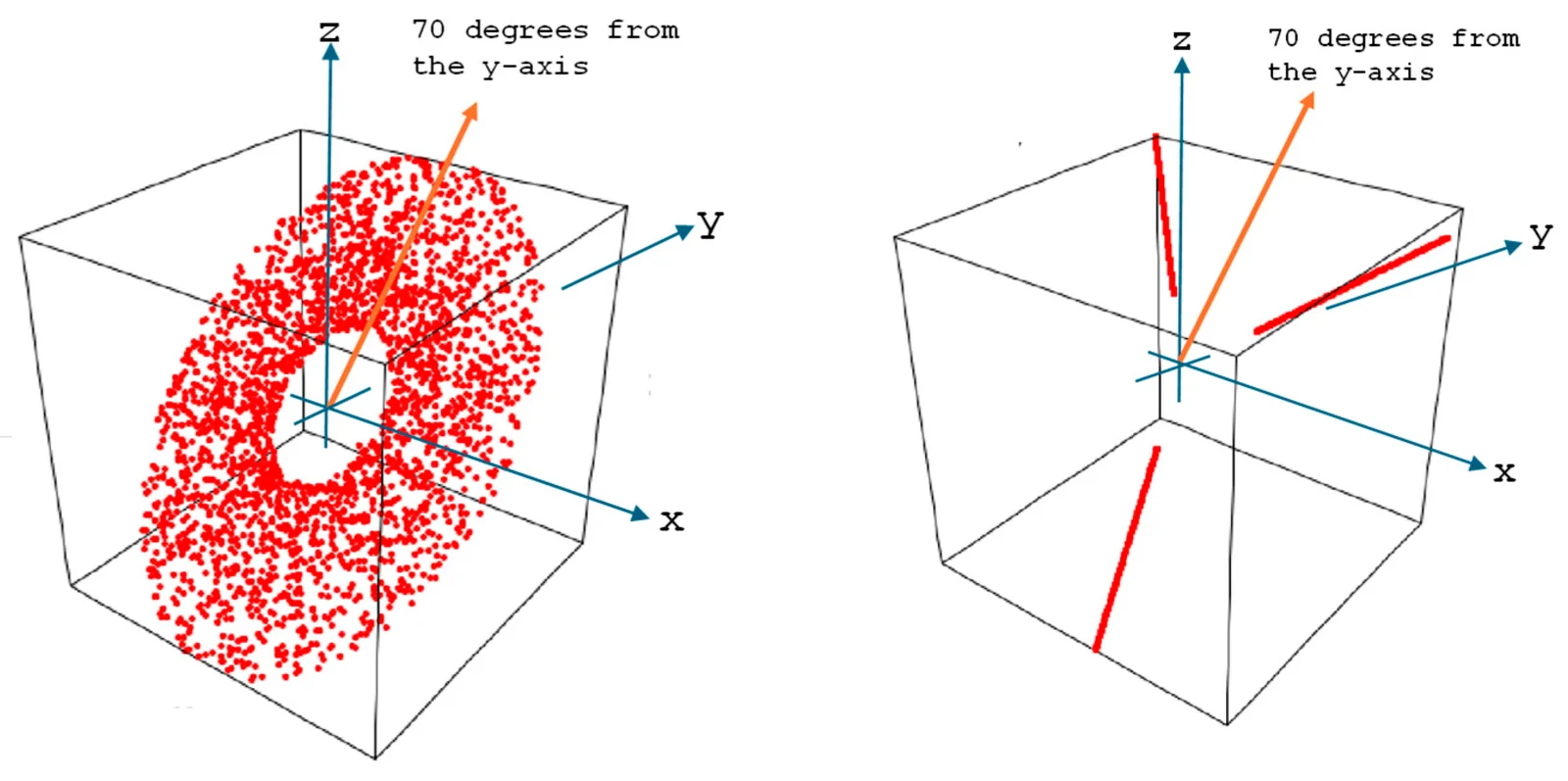
Two set of 3D points for simulation.

Figure 5 shows two sets of simulation data for convolution-based HALF calculation, each of which lies on a plane and on lines to validate the aforementioned prospect through mathematical convolution. These sets are inclined at an angle of 70 degrees from the y-axis. A critical aspect of this prospect is to neglect the thickness of any real objects. In the data, each point $p={\left(x,y,z\right)}^{T}$ has its phase angle ${\eta =\tan}^{-1}\left(\frac{e_{x}^{T}p}{\left|p\right|},sign\left(e_{x}\times p\right)\left|e_{x}\times p\right|\right)$ measured from the origin line $e_{x}={\left(1,0,0\right)}^{T}$ for calculating the fundamental histogram of phase angles.

**Figure 6:**
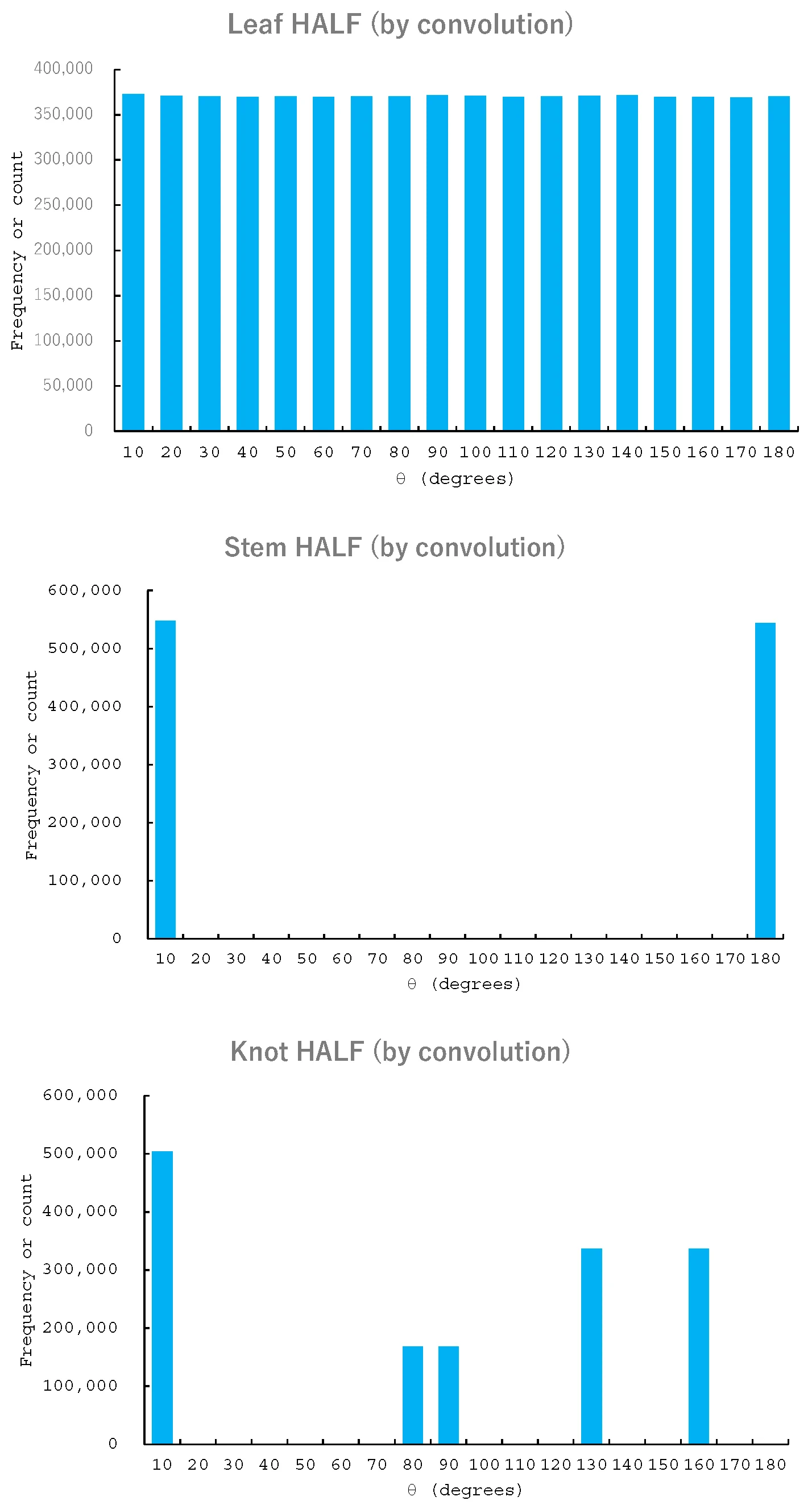
Typical HALFs by convolution-based calculation.

Figure 6 presents clear profiles of histograms for three classes of plant parts: leaf, stem, and knot, created through the previously mentioned mathematical formalization. The histogram for the leaf class shows a uniform distribution, while the stem class exhibits a bi-modal histogram. The histogram for the knot class features multiple peaks, in this case four, with one peak splitting into two due to trivial numerical quantization around 80 or 90 degrees.

It is important to note that the condition for understanding our HALF calculation as an extension of convolution is not always satisfied for any real shapes of plants in PCD. However, the mathematical framework for constructing HALF is clear and effective for gaining insights into the HALF structure based on real data.

### 3.3. Similarity Evaluation in HALF

To design effective discrimination or classification methods, an effective similarity measure based on HALF is essential. The intersection is utilized to obtain a simple similarity between any two histograms of the same size, which can prove to be an effective approach [23,24]. In the aforementioned HALF analysis, any HALF is represented as a $\hat{p}$-tuple of ordered positive integers or real numbers, $H={\left\{f_{j}\right\}}_{j=1,\ldots ,p}$. In this paper, we define three classes of plant parts: leaf, stem, and knot, each with its own reference HALF, $G={\left\{g_{i}\right\}}_{i=1,2,\ldots ,p}$. For each pair of $\left(H,\;G\right)$, the intersection is defined as follows:

$$\zeta =\sum_{i=1}^{p}min\left\{f_{i},\;g_{i}\right\}$$

An ordinary scheme for discrimination is as follows: based on the maximum value of ζ, the interest point $p_{0}$ is classified into the *k* class, similar to linear discrimination rules.

**Figure 7:**
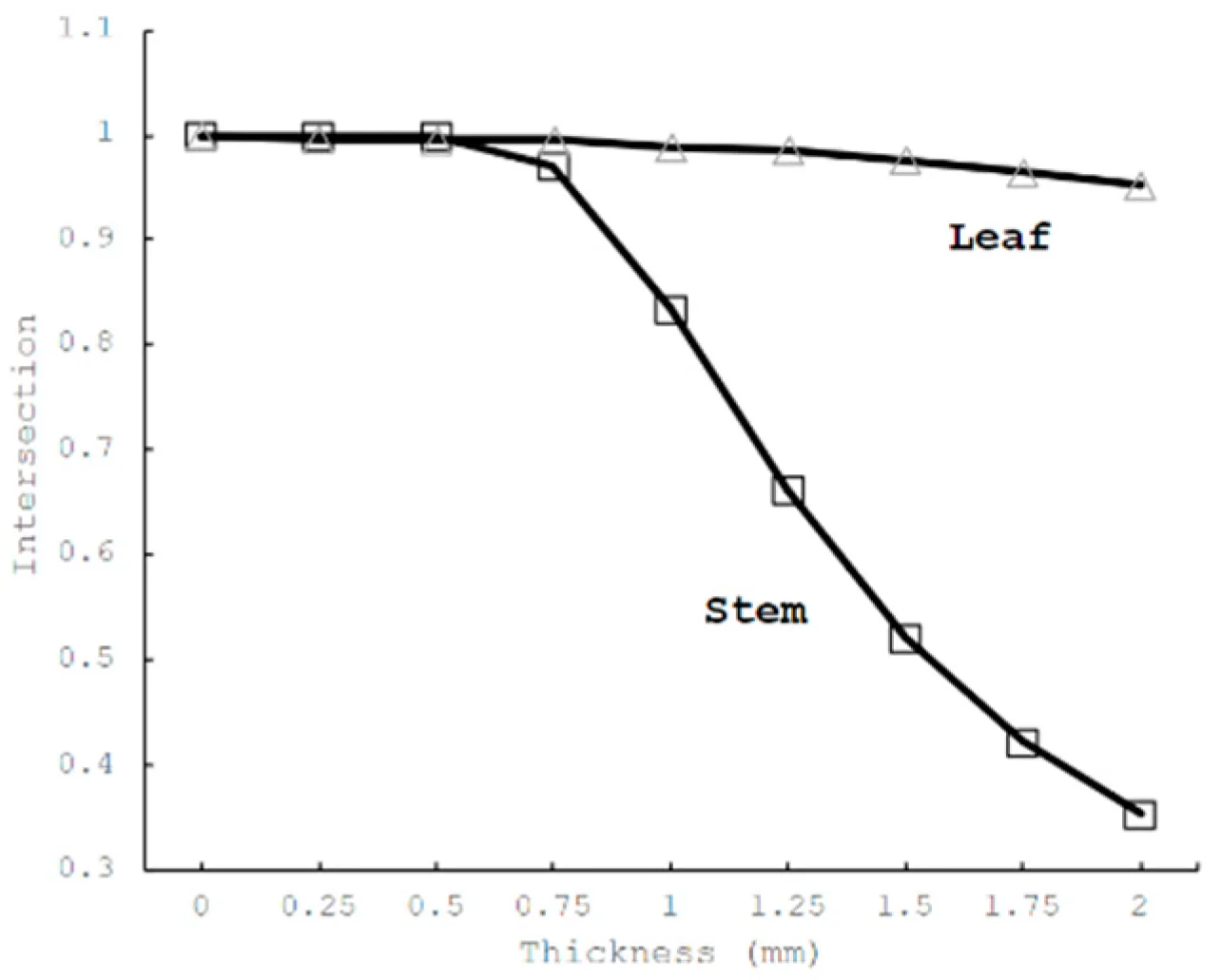
Relationship between leaf and stem thickness and the HALF degradation trend.

As shown in the previous section, the calculation of distribution based on convolution is a mathematically rigorous scheme for HALF. For plant structure analysis, there may be advantages in analyzing thin leaves and stems. Here, we examine the limitations of HALF analysis when applied to more realistic 3D objects. Figure 7 illustrates the results of a simulation experiment using a thicker leaf and stem, modified from the objects shown in Figure 5. The differences between them and the ideal HALF were calculated using histogram intersection.

From this figure, we observe that leaf HALFs remain effective for diameters between 0 and 2 mm, whereas stem HALFs degrade in quality as their thickness increases. Notably, in this experiment stems with diameters over approximately 1 mm lost their similarity to the ideal stem with no diameter. This occurred because stem HALFs generally exhibit peaks in very narrow regions around 0 and 180 degrees, so the introduction of thicker diameters caused a significant increase in variance around these peaks. This phenomenon may also occur in knot HALFs. The reason for the endurance of quality in leaf HALFs may relate to the good uniformity of frequency within them; thus, the introduction of thinness does not have a severe effect on their HALFs. This consideration may be necessary for HALF-based algorithms handling more complicated shapes, such as roots, fruits, or flowers.

## 4. Sequential Competitive Segmentation Algorithm: SCSA

### 4.1. 3D Point Cloud Data for Soybean Plant

We planted the soybean variety GmJMC112 (FUKUYUTAKA), which was derived from the Genebank project at NARO (https://www.gene.affrc.go.jp), accessed on 15 April 2025. The seed was sown in a 1/5000a Wagner pot in 2018 using baked soil, and the plant was grown in the greenhouse at the Kazusa DNA Research Institute. A water tube was installed in the pot, with irrigation occurring twice a day. The 3D point cloud data was obtained by capturing all-surrounding images in a studio where the cultivation pot is rotated in front of the camera and then reconstructing the 3D point cloud using the photogrammetry method.

### 4.2. Definition of Optimum Reference HALFs

A prominent feature of growing plants are variations in the sizes and shapes of their parts as they grow. These characteristics can be observed even in individuals from the same epoch. For instance, the shapes of knots in the lower parts of a plant may differ significantly from those in the upper parts, particularly in thickness. Conducting research on living plants can present challenges. In this paper, we explore a technical trial using an original approach to segment or classify plant parts into optimal classes using the optimal reference HALF, which are sufficiently distinct from each other to facilitate similarity evaluation.

**Figure 8:**
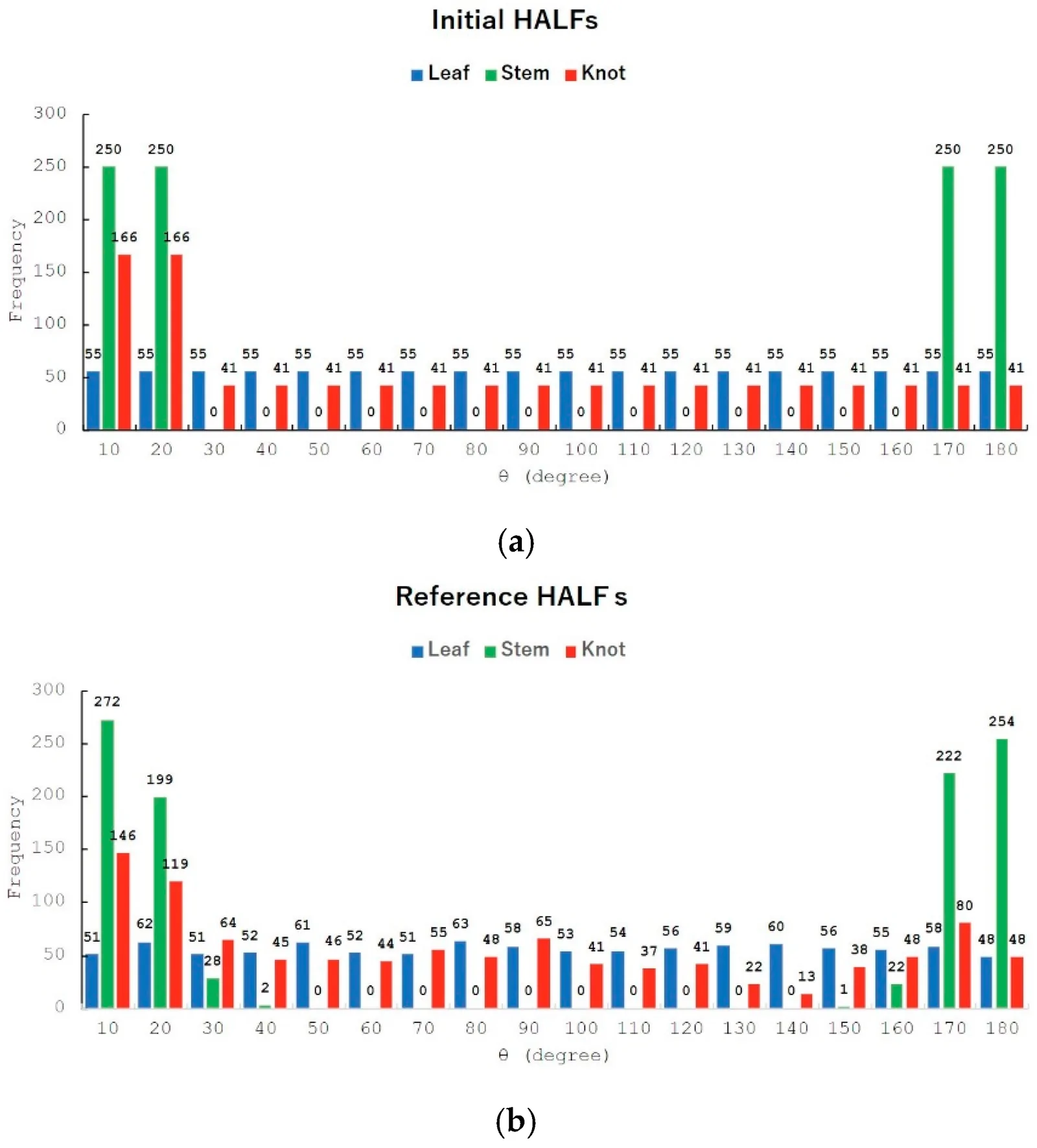
Initial and optimum reference HALFs. (**a**) Initial HALFs. (**b**) Optimum reference HALFs detected using initial HALFs.

Figure 8 shows the initial HALFs and the detected optimum HALFs for three classes. Any initial HALFs can be defined through an approach based on convolution.

### 4.3. SCSA and Elemental Procedures

**Figure 9:**
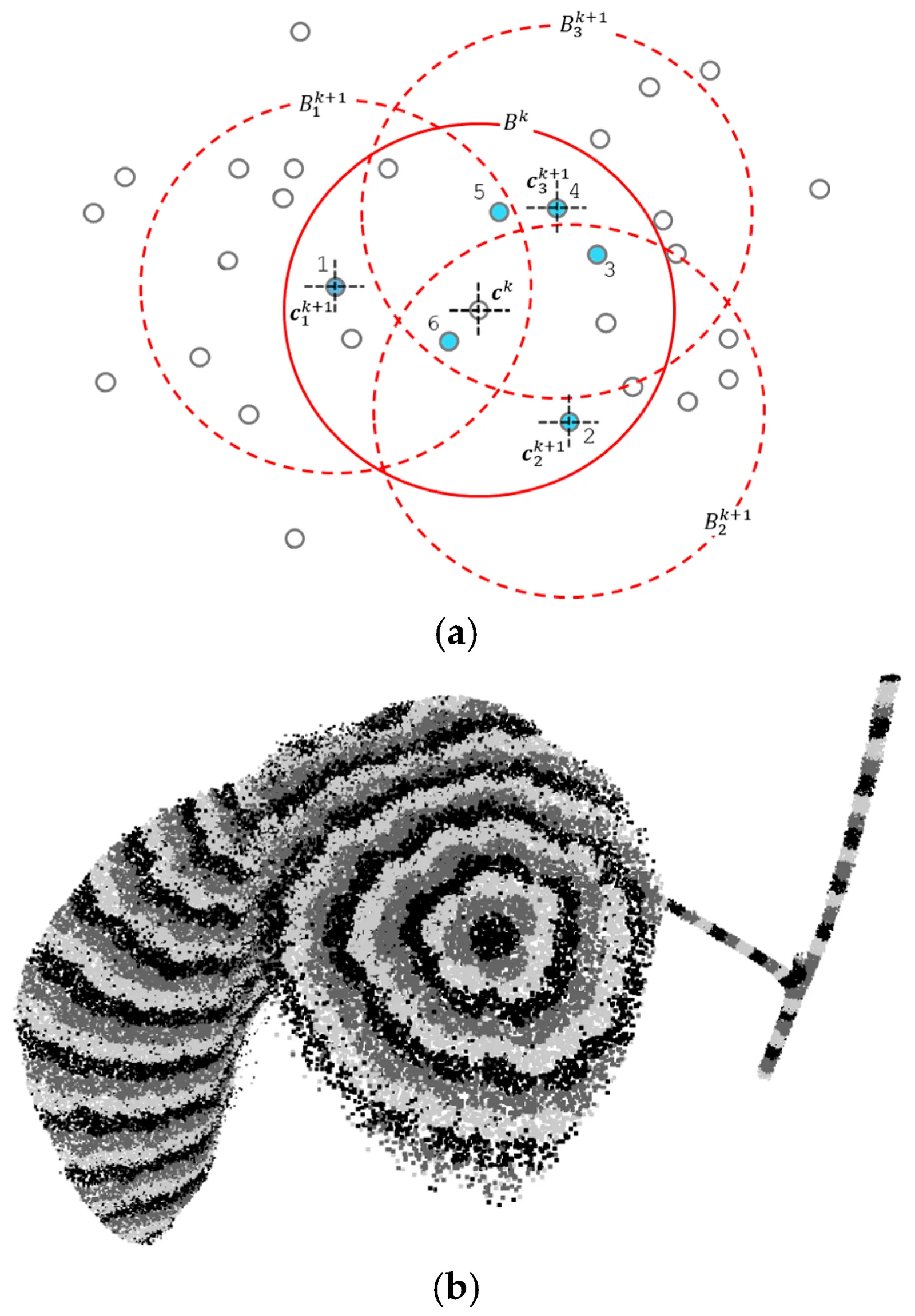
Small example of the SCSA process. (**a**) Bubble-based selection algorithm (BSA) used in sequential extensive merging. Bubbles define segmentation points for the selection process. (**b**) Example of applying BSA with uniformly distributed bubbles. For 131,641 data points, 61 iterations were completed (r=5 mm).

Figure 9a shows a snapshot at the k + 1st iteration of Bubble-based Selection Algorithm (BSA) used in sequential extensive merging, where a bubble radius r, centered at the segmented point $c^{k}$ from the previous iteration, $B^{k}=\left\{p;\left\lfloor p-c^{k}\right\rfloor \leq r\right\}$, is utilized to enclose PCDs for segmentation. The point $c^{k}$ is classified into the best class according to the current reference HALFs at that point in time. In this example, six points colored blue are labeled as belonging to the same class as $c^{k}$ and are then sorted by their distances from $c^{k}$. Initially, after defining or segmenting $p_{1}$ as $c_{1}^{k+1}$, a new bubble $B_{1}^{k+1}=\left\{p;\left\lfloor p-c_{1}^{k+1}\right\rfloor \leq r\right\}$ is created around it. Next, since $p_{2}$ is not included in $B_{1}^{k+1}$ and there are not enough segmented points yet, this $p_{2}$ defines the next segmented point $c_{2}^{k+1}$ and creates a new bubble $B_{2}^{k+1}$. The points $p_{3}$, $p_{5}$, and $p_{6}$ do not contribute to segmentation because they are involved in $B_{1}^{k+1}$ or $B_{2}^{k+1}.$ Lastly, $p_{4}$ satisfies the condition to be considered a new segmented point $c_{3}^{k+1}$. In this experiment, we set a limitation on extending new segmentation to just four points surrounding $c^{k+1}.$ Figure 9b illustrates a small-scale real example of SCSA processing. Notably, the uniform extension of bubbles for two classes—leaves and stems—was maintained throughout the SCSA process.

Table 1 presents the BSA process from the perspective of distance evaluation between seed points, $d_{ij}=\left|p_{j}-c_{i}^{k+1}\right|$, where the matrix $D=\left(d_{ij}\right)$ represents the sequence of segmentation.

### 4.4. Stepwise Details of SCSA

The following steps constitute a segmentation process based on the BSA scheme described in Figure 9 in the previous section.

SCSA in steps:

Initialization:

Step 1. Set candidate seed points (CSPs) for leaf, stem, and knot classes that satisfy two conditions: a CSP must have over 85% similarity to the reference HALF, indicating good quality, and must be at least 24 mm distant from any other CSPs in the same class. The threshold of 85% was empirically chosen based on testing of nearby values.

Phytomer segmentation:

Step 2. Select four SPs for the leaf, upper and lower stems, and knot from the CSPs defined in Step 1. These SPs should have connectivity as defined by the bubble-based selection algorithm shown in Figure 9a. The lower stem SP should be lower in height than the knot SP due to the shape of the phytomer structure.

Step 3. Using BSA, select and competitively compare SPs to define segmentation or classification into three classes through an alternate width-priority search.

Step 4. Return any already classified CSPs for leaves and stems, except for knots, back to the pool of normal points.

Repeat Steps 2 through 4 while any CSP for leaves remains.

Stem segmentation:

Step 5. Select the best SP of good quality for the stem. Then select another CSP for the knot that exhibits connectivity just as in Step 2.

Step 6. Using BSA, select and competitively compare SPs to define segmentation or classification into two classes through an alternate width-priority search.

Step 7. Return any already classified CSPs for stems, except for knots, back to the pool of normal points.

Repeat Steps 5 through 7 while any stem CSP remains.

**Figure 10:**
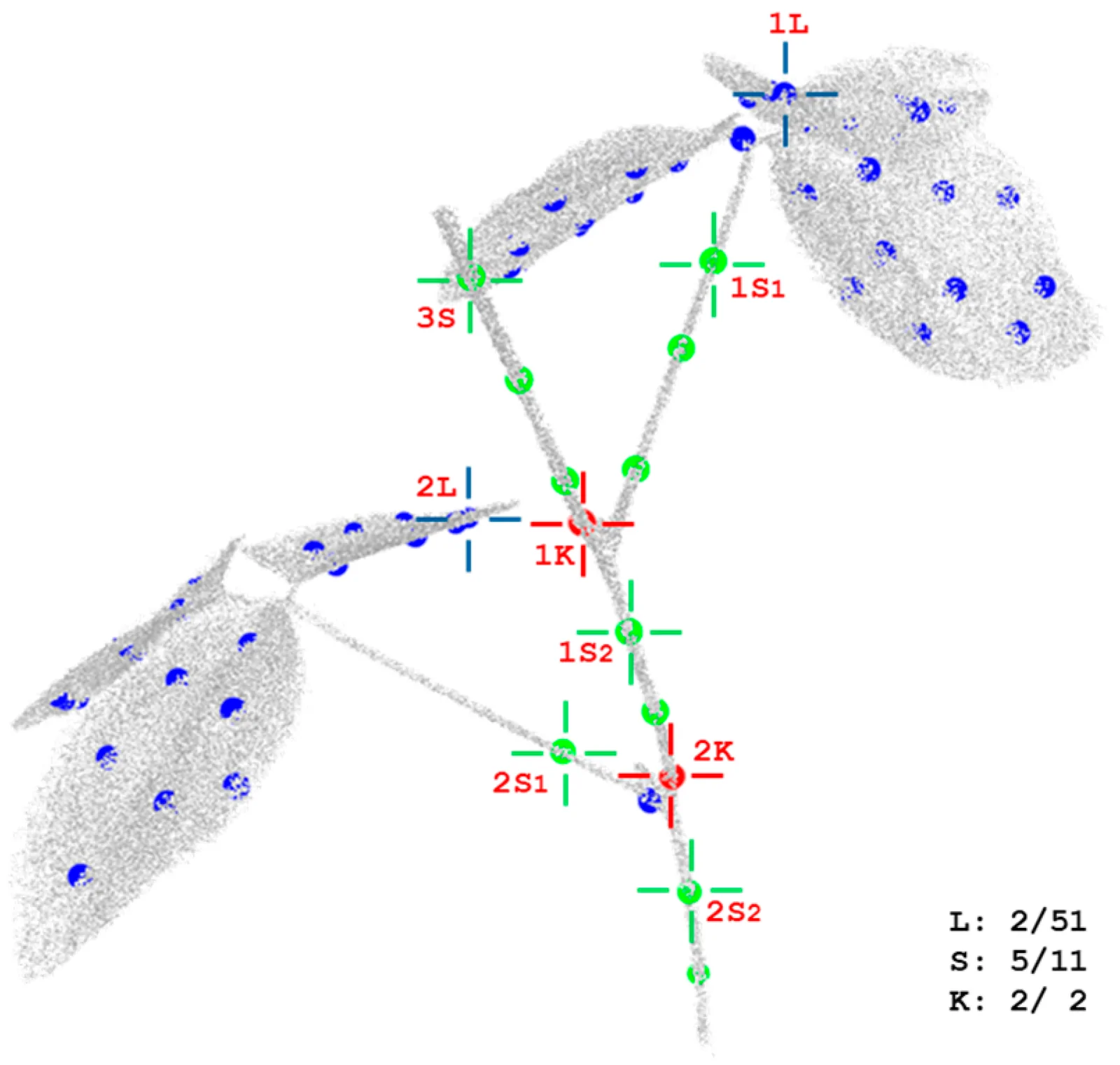
Candidate seed points for leaf (blue), stem (green), and knot (red) classes.

Figure 10 represents a set of candidate seed points defined in Step 1 of the SCSA, consisting of 51, 11, and two candidate seed points for the three classes. The red points indicate the round number of phytomer segmentation and the corresponding class ID. The SPs, S_1_ and S_2_, represent the upper and lower stem seeds, respectively.

**Figure 11:**
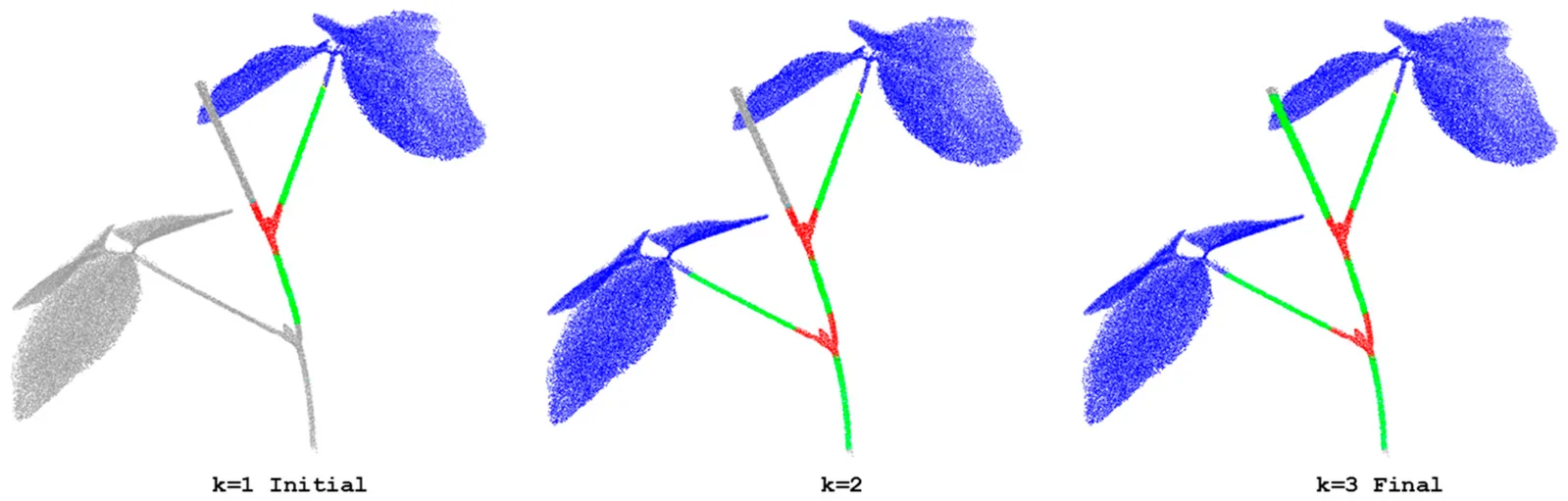
SCSA segmentation of two phytomers. Gray points represent data that have not yet been classified.

Figure 11 shows the results of phytomer segmentation using the SCSA. In the first round of phytomer segmentation (Steps 2 through 4), the right-hand upper phytomer, which consists of the leaf-stem-knot-stem structure, was successfully segmented and extracted; subsequently, the left-hand lower phytomer was recognized, as shown in the figure. Finally, in the third round (Steps 5 through 7), the upper center region was identified as a stem.

### 4.5. Segmentation Performance Evaluation

**Figure 12:**
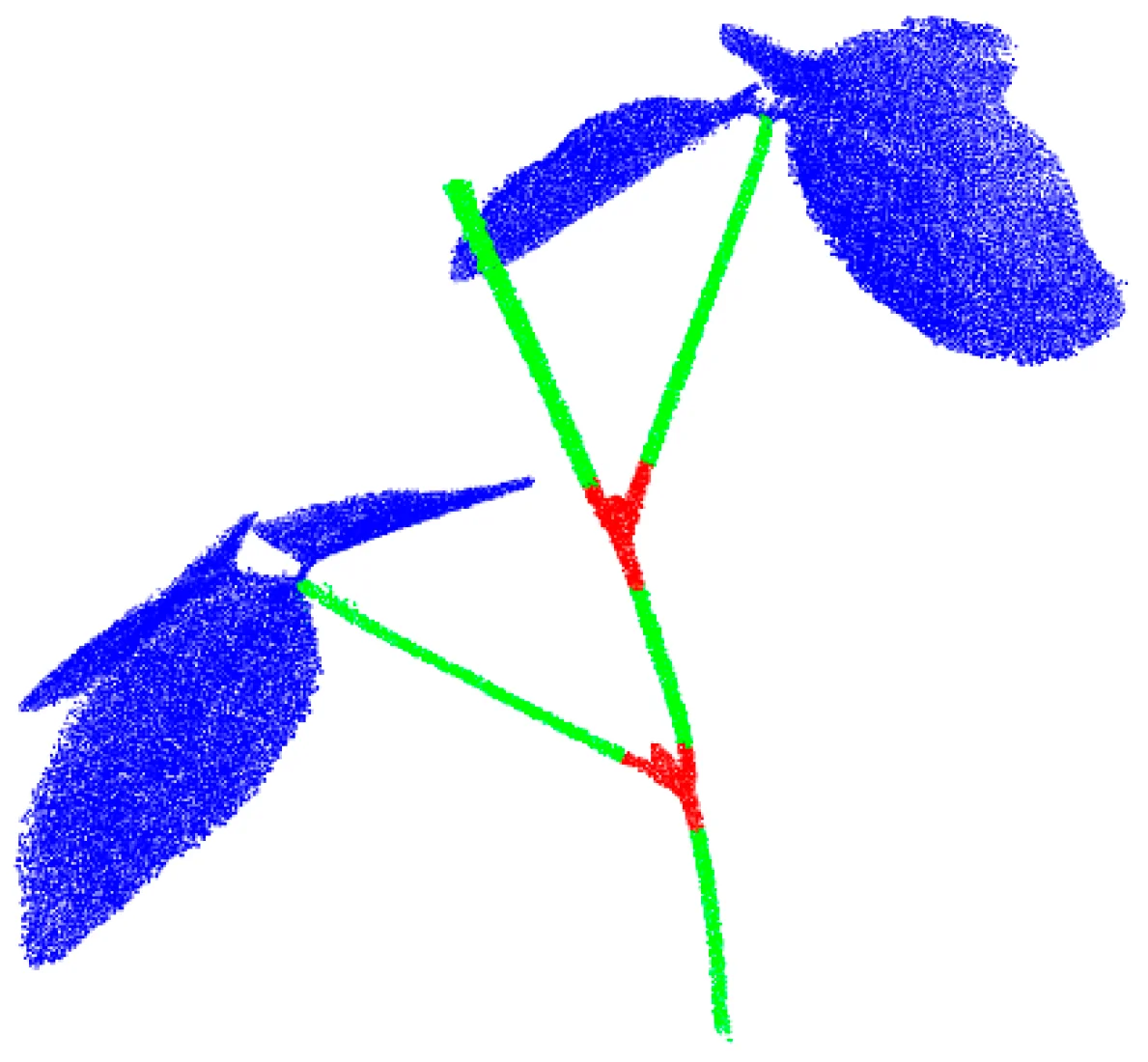
Ground -truth segmentation.

The segmentation performance of the SCSA was evaluated by comparing it with the ground-truth segmentation. Figure 12 shows the ground-truth segmentation, which was created by manually labeling each point in each class as either a leaf, a stem, or a knot. Table 2 presents the total number of point clouds in the ground truth, as well as the number of point clouds in each class. Determining the boundary between the knot and the stem is challenging when labeling the ground truth. Therefore, the center of the knot was manually specified, and a group of points within a sphere with a radius five times the stem diameter was classified as a knot. This setting also classifies the stipules as knots.

Recall, precision, and F-score were utilized as metrics for accuracy evaluation. Table 3 presents the evaluation results for each class. The leaf class showed better performance than the other classes, as the number of points in the leaf class is large and the effect of misclassification is minimal. The low recall of the stem class is attributed to misclassification at its boundary with the leaf class and the influence of unclassified point clouds at the stem tip. The lower evaluation results of the knot class compared to the other classes suggest that its boundary with the stem class is unclear. In particular, the low precision of the knot class may also be influenced by class imbalance, as the knot class accounts for only 1.6% of the dataset. The comparison with the ground truth confirms that SCSA is less prone to misclassification, except at the endpoints of the region, due to its use of a region-growing method.

All computations were performed on a machine running Windows 11 Home, equipped with an Intel(R) Core(TM) i9-12900H CPU (2.50 GHz) and 16.0 GB of RAM. The processor was manufactured by Intel Corporation, headquartered in Santa Clara, CA, USA. The HALF feature extraction process required approximately 27.0 s to process a point cloud with 136,502 points, resulting in an average computation time of approximately 0.198 milliseconds per point. The SCSA segmentation process took approximately 2.25 s. The total computation time, averaged over 100 runs, was approximately 29.3 s. Since further reductions in computation time may be necessary, the proposed HALF-SCSA method is currently considered suitable for offline use.

### 4.6. Comparison with Existing Method

**Figure 13:**
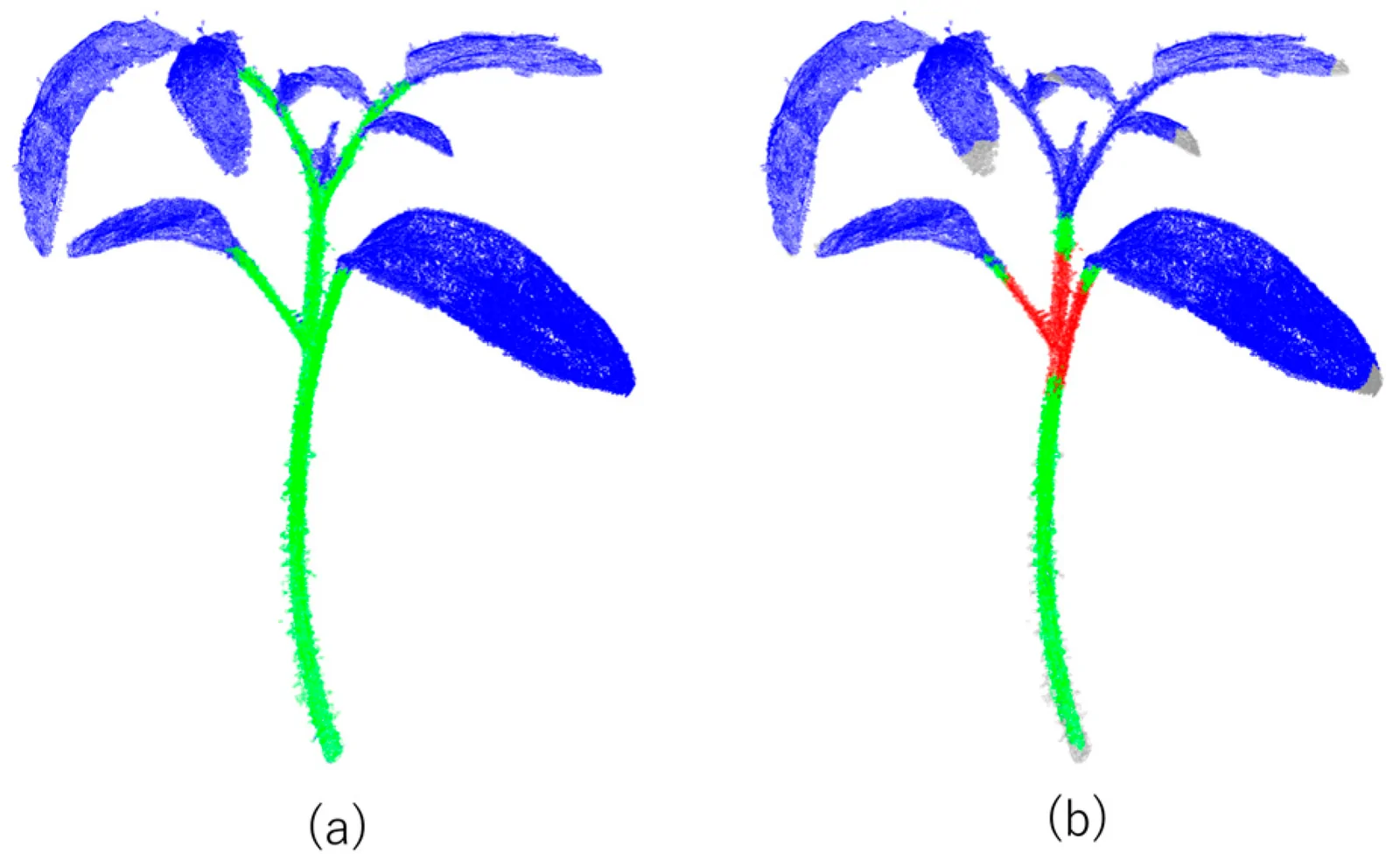
Comparison of segmentation results on tomato point cloud data using Plant 3D (P3D) and the proposed HALF-SCSA. (**a**) P3D result using a pre-trained model: leaf and stem classification. (**b**) HALF-SCSA result: segmentation into leaf, stem, and knot.

To further evaluate the proposed HALF-SCSA method, we conducted an additional experiment using tomato 3D point cloud data and the Plant 3D (P3D) toolkit [8], which classifies points into leaf and stem categories based on FPFH [7] features and a trained neural network model. In this experiment, we used the pre-trained model provided by P3D without retraining. Figure 13 shows the visual comparison between the segmentation results of P3D and HALF-SCSA. As a result, P3D produced binary segmentation distinguishing only leaf and stem regions. In contrast, HALF-SCSA segmented the same data into three categories: leaf, stem, and knot. While the difference in the number of output classes reflects the design of the models used in this comparison, we emphasize that HALF-SCSA achieved this segmentation without any training or labeled data, highlighting its practical advantage as a learning-free and easily transferable method. However, as can be seen in Figure 13, although the lower knot was successfully segmented, the upper knot was not clearly identified. This suggests that further refinement may be needed to improve the method’s robustness in capturing diverse structural characteristics across different plant types.

**Figure 14:**
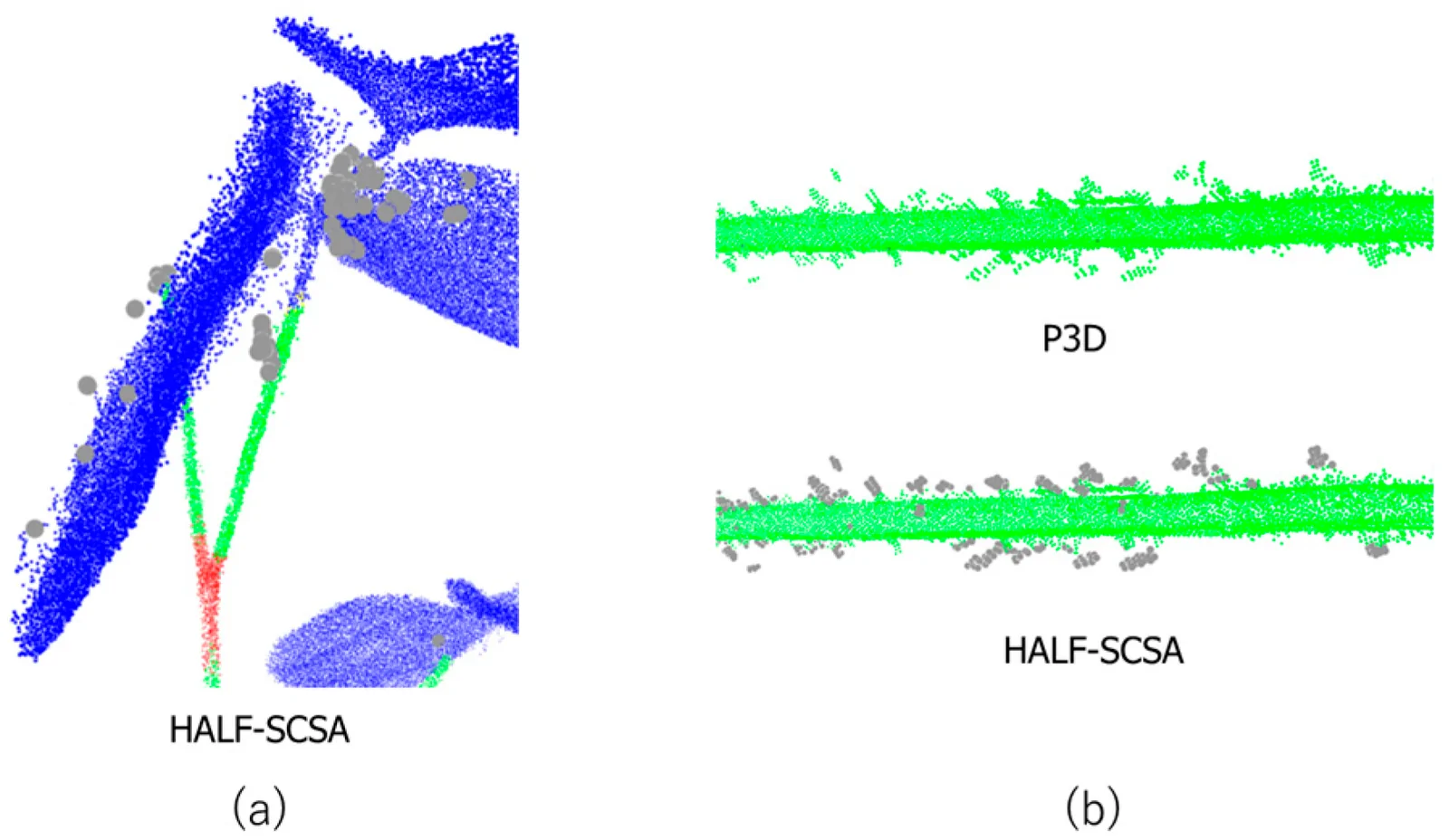
Examples of noisy points. (**a**) HALF-SCSA result on soybean data (gray points are unclassified). (**b**) Comparison between P3D (top) and HALF-SCSA (bottom) on tomato data.

Furthermore, we also investigated how the proposed method handles noise (including outliers), which is a common and significant issue in plant phenotyping. Figure 14 shows regions in the soybean and tomato datasets where noisy points were observed. In (a), HALF-SCSA treats outliers as unclassified (gray) points which are visually enhanced for clarity. In (b), HALF-SCSA excludes noisy points by labeling them as unclassified, whereas P3D assigns all points to the stem class. This behavior is attributed to the design of the SCSA, which locally evaluates spatial continuity and avoids assigning unreliable labels to ambiguous points, thereby reducing misclassification. Although this evaluation is qualitative, it demonstrates that the proposed method exhibits a certain degree of robustness to real-world noise.

### 4.7. Limitation

The proposed HALF-SCSA method was designed for plants with clearly distinguishable organ structures, such as leaves, stems, and knots, as typically found in soybean. Soybean is an agriculturally important crop, and detailed morphological analysis of its organs is critical for crop breeding and cultivation management. The success of HALF-SCSA in tomato, which exhibits similar morphological traits, suggests its potential applicability to other crops with comparable organ structures. However, for plants with significantly different overall architecture and organ shapes—such as rice—the current method may not be directly suitable. We recognize this as a limitation of our approach. Future work will explore algorithmic extensions and alternative feature representations to accommodate a broader range of plant morphologies.

## 5. Conclusions

In this study, we proposed a novel segmentation method, HALF (Histogram of Angles in Linked Features), for analyzing plant morphology using 3D point cloud data. This method utilizes local angular information within 3D point clouds as statistical features, enabling the identification of key plant structures (leaves, stems, and knots) without requiring labeled data. Furthermore, we introduced the Sequential Competitive Segmentation Algorithm (SCSA), which enables phytomer-level classification using HALF. The effectiveness of HALF was confirmed through evaluation experiments using soybean 3D point cloud data, demonstrating the feasibility of SCSA-based classification.

Since HALF directly processes 3D point cloud data acquired through sensing technologies such as LiDAR, laser scanning, and photogrammetry, it is well-suited for integration into sensor-based plant monitoring systems, making it highly applicable to plant phenotyping tasks. This capability also adds value to high-throughput phenotyping, precision agriculture, and the development of advanced sensing platforms for automated plant analysis.

Future research will focus on extending the applicability of HALF to enable more detailed plant morphological analysis. As part of this effort, we aim to develop a method for detailed leaf segmentation, distinguishing specific structures such as leaf edges and leaf tips. Additionally, we will expand the application of HALF beyond segmentation to the quantitative measurement of plant morphology. By utilizing HALF for precise growth analysis and cultivar evaluation, we aim to enhance the accuracy of 3D plant structure analysis and broaden its practical applications.

## Figures and Tables

**Table 1:** BSA from the perspective of distance.

D		$p_{j}$					
		1	2	3	4	5	6
$c_{i}^{k+1}$	1	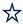	$d_{12}$	$d_{13}$	$d_{14}$	$d_{15}$	$d_{16}$
	2		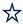	$d_{23}$	$d_{24}$	–	–
	3				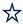	–	–
		$B_{1}^{k+1}$	$B_{2}^{k+1}$		$B_{3}^{k+1}$		

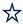
 shows segmented points at the center of the bubbles.

**Table 2:** Number of point clouds in ground-truth segmentation.

Total	Knot	Stem	Leaf
136,502	2194	7191	127,117

**Table 3:** Segmentation performance of SCSA.

	Precision (%)	Recall (%)	F-Score (%)
Leaf	99.6	99.9	99.8
Stem	96.5	84.1	89.9
Knot	78.7	89.8	83.9

## Data Availability

The raw data supporting the conclusions of this article will be made available by the authors on request.
